# Altered static functional network connectivity predicts the efficacy of non-steroidal anti-inflammatory drugs in migraineurs without aura

**DOI:** 10.3389/fnmol.2022.956797

**Published:** 2022-09-13

**Authors:** Heng-Le Wei, Wen-Juan Yang, Gang-Ping Zhou, Yu-Chen Chen, Yu-Sheng Yu, Xindao Yin, Junrong Li, Hong Zhang

**Affiliations:** ^1^Department of Radiology, Nanjing Jiangning Hospital, Nanjing, China; ^2^Department of Neurology, Nanjing Jiangning Hospital, Nanjing, China; ^3^Department of Radiology, Nanjing First Hospital, Nanjing Medical University, Nanjing, China

**Keywords:** migraine, functional network connectivity, non-steroidal anti-inflammatory drugs, efficacy, machine learning

## Abstract

Brain networks have significant implications for the understanding of migraine pathophysiology and prognosis. This study aimed to investigate whether large-scale network dysfunction in patients with migraine without aura (MwoA) could predict the efficacy of non-steroidal anti-inflammatory drugs (NSAIDs). Seventy patients with episodic MwoA and 33 healthy controls (HCs) were recruited. Patients were divided into MwoA with effective NSAIDs (M-eNSAIDs) and with ineffective NSAIDs (M-ieNSAIDs). Group-level independent component analysis and functional network connectivity (FNC) analysis were used to extract intrinsic networks and detect dysfunction among these networks. The clinical characteristics and FNC abnormalities were considered as features, and a support vector machine (SVM) model with fivefold cross-validation was applied to distinguish the subjects at an individual level. Dysfunctional connections within seven networks were observed, including default mode network (DMN), executive control network (ECN), salience network (SN), sensorimotor network (SMN), dorsal attention network (DAN), visual network (VN), and auditory network (AN). Compared with M-ieNSAIDs and HCs, patients with M-eNSAIDs displayed reduced DMN-VN and SMN-VN, and enhanced VN-AN connections. Moreover, patients with M-eNSAIDs showed increased FNC patterns within ECN, DAN, and SN, relative to HCs. Higher ECN-SN connections than HCs were revealed in patients with M-ieNSAIDs. The SVM model demonstrated that the area under the curve, sensitivity, and specificity were 0.93, 0.88, and 0.89, respectively. The widespread FNC impairment existing in the modulation of medical treatment suggested FNC disruption as a biomarker for advancing the understanding of neurophysiological mechanisms and improving the decision-making of therapeutic strategy.

## Introduction

Migraine is a persistent, disabling neurological disorder involving moderate-to-severe headache attacks, which can last up to 72 h, and related unpleasant symptoms (Headache Classification Committee of the International Headache Society [IHS], 2013). It is the second leading cause of neuronal disability in the world, which may lead to personal suffering and impaired quality of life with significant socioeconomic burdens (Feigin et al., 2019; Abrams et al., 2020). In the United States alone, its estimated healthcare cost is about $1 billion annually (Mennini et al., 2008). Migraine affects approximately 15% of the general population worldwide (Abrams et al., 2020). Given the health and socio-economic burden caused by migraine, exploring effective treatments for the disorder is one of the key areas of clinical research. Several studies have continuously proposed, developed, and tested more specific drugs for migraine treatment. Although several pharmacological choices are available to treat migraines, none of such treatments are ideal for most people. Regardless of that, non-steroidal anti-inflammatory drugs (NSAIDs) are still the first-line drugs for the acute treatment of migraines (Ashina, 2020). Currently, the selection strategy for NSAIDs is still mainly based on trial and error. Possible explanations include various pathophysiological mechanisms with complexity in migraine. The trial-and-error approach not only prolongs the treatment time but also increases the ineffective cost. Recent studies have focused on understanding the pathophysiological mechanism of migraine to develop new theories for improving the treatment efficacy. For instance, neuroimaging studies have transformed the understanding of migraine from a vascular to a neurovascular, and most recently, to a central neural system disorder (Schwedt et al., 2015). However, the effect of brain functional alterations on migraine treatment remains unknown.

The resting-state functional magnetic resonance imaging (rs-fMRI), a non-invasive neuroimaging examination, has therefore attracted considerable attention. It is reported that neurogenic inflammation resulting from activation of the trigeminovascular pathway is the main cause of migraine attacks (Goadsby et al., 2017). Various studies have suggested that the cortical feed-forward system originates from the trigeminal neurovascular pathway and connects to the higher-sensory cortex (Brennan and Pietrobon, 2018; Tu et al., 2020). Yu et al. (2017) and Chen et al. (2019) revealed the alterations in either regional brain activity or functional connectivity among core cognitive networks located in the trigeminovascular pathway, such as the default mode network (DMN), executive control network (ECN), and salient network (SN), and their correlations with clinical characteristics in patients with migraine. Nonetheless, they only focused on regional and seed-based functional changes, or a limited number of prior selected networks, and have not done a comprehensive analysis of the abnormal connections among the large-scale functional networks in migraine. Some studies have linked complex subjective experiences like perception and processing of pain in patients with migraine to functional integration among intrinsic large-scale functional networks and reported significant differences (Zhang et al., 2016; Coppola et al., 2018; Wei et al., 2020). These studies provided initial evidence regarding abnormal interactions among the functional networks in migraine. Although these rs-fMRI studies have proved that functional alterations between different large-scale networks were associated with the neurophysiological mechanism of migraine, less is known about the interactions among the large-scale networks and their influence on the treatment efficacy in migraine.

Additionally, machine learning models based on clinical and neuroimaging data have shown great potential in constructing automatic predictors in the field of classification and treatment of migraine (Pérez Benito et al., 2019; Kwon et al., 2020; Liu et al., 2020; Mu et al., 2020). However, predictors for the efficacy of NSAIDs are still in their infancy. It has been revealed that migraine subtypes can have various influences on the efficacy of drugs because of their diverse pathophysiological mechanisms. Therefore, this study attempted to investigate the possible neural mechanisms for underpinning the efficacy of NSAIDs in patients with migraine without aura (MwoA) using a combination of functional network connectivity (FNC) analysis and a support vector machine (SVM) algorithm. We hypothesized that patients with MwoA and showing an effective response to NSAIDs (M-eNSAIDs) may display significant FNC differences among large-scale networks involved in modulating nociception and predicting efficacy, compared to patients with ineffective response to NSAIDs (M-ieNSAIDs). Substantive differences, as revealed in this study, may be linked with clinical characteristics, and provide novel insights into elucidating the pathophysiological mechanism and developing treatment strategies for migraine.

## Materials and methods

### Participants

A total of 73 patients diagnosed with episodic MwoA in the neurological outpatient clinic were prospectively and continuously enrolled. The diagnosis of patients with MwoA was based on the International Classification of Headache Disorders, 3rd edition (ICHD-3) (Headache Classification Committee of the International Headache Society [IHS], 2013). To control the potential pharmacological and physiological effects, the inclusion criteria were: (1) patients were drug-free for at least 1 month before being enrolled; (2) patients in the interictal phase were headache-free for at least 3 days before and after scanning, ascertained by a structured telephonic interview. Thirty-three healthy subjects (all right-handed) matched for age, sex, and education level were recruited as healthy controls (HCs). The general exclusion criteria included: (1) comorbidity with other forms of headache and neuropsychological or neurological diseases, (2) a history of previous brain injury or psychoactive medication use, (3) a history of alcohol or drug abuse, (4) pregnant or lactating women, and (5) any contraindications to MRI scanning.

### Clinical characteristics

All migraine participants underwent comprehensive questionnaires during the first visit and were followed up via telephone. The questionnaires primarily include demographic data (e.g., age, sex, and education level) and migraine characteristics (e.g., disease duration, frequency and attack duration, headache intensity, the extent of the impact, and burden on quality of life). Namely, headache intensity was recorded on the Visual Analog Scale (VAS); impact and disability on an individual were assessed by Headache Impact Test-6 item (HIT-6) and Migraine Disability Assessment Scale (MIDAS), respectively. We also applied the Montreal Cognitive Assessment (MoCA) to evaluate cognitive impairment (all participants’ MoCA scores were > 25). Patients were asked to record a headache diary about headache intensity (VAS score) before and 2 h after drug intake within 3 months after scanning. According to the criteria, the complete response, partial response, minimal response, and no response were classified as > 75% reduction, 50–75% reduction, 25–50% reduction, and < 25% reduction in VAS scores, respectively (Yadav et al., 2020). The response to NSAIDs was defined as a 50% or greater reduction in pain intensity from pre-treatment level to post-treatment level at least 2 times.

### Magnetic resonance imaging acquisition

All MRI data were acquired on a 3.0-Tesla Philips MRI scanner (Ingenia) with an eight-channel head coil. For this analysis, the functional images were acquired axially using a gradient echo-planar imaging sequence as follows: repetition time (TR) = 2,000 ms; echo time (TE) = 30 ms; slices = 36; thickness = 4 mm; gap = 0 mm; field of view (FOV) = 240 mm × 240 mm; acquisition matrix = 64 × 64; and flip angle (FA) = 90°. Moreover, structural images were obtained using a three-dimensional turbo fast echo T1WI sequence with the following parameters: TR/TE = 8.1/3.7 ms; slices = 170; thickness = 1 mm; gap = 0 mm; FA = 8°; acquisition matrix = 256 × 256; FOV = 256 mm × 256 mm. During the scanning, scanner noise and head motion were reduced using earplugs and foam padding, and the participants were instructed to reflex and lie with their eyes closed but not fall asleep.

### Data pre-processing

Image preprocessing was performed using the Resting-state fMRI Data Analysis Toolkit plus V1.24 (RESTplus V1.24).^1^ The preprocessing included the following steps: discarding the first 10 volumes, slice timing correction with the 35th slice as the reference, realignment of head motion, and normalizing corrected images to the Montreal Neurological Institute space (3 × 3 × 3 mm^3^) using the diffeomorphic anatomical registration through exponentiated lie (DARTEL) algebra, and spatial smoothing with a 6-mm full-width half-maximum (FWHM) Gaussian kernel. Moreover, subjects with excessive head motion in any direction (>2 mm or 2°) were excluded from the analysis.

### Independent component analysis and static functional network connectivity analysis

The Group ICA of fMRI Toolbox (GIFT 4.0a)^2^ was used to extract independent networks. The preprocessed data were automatically decomposed into spatial independent components (ICs) by the minimum description length (MDL) criteria. Then the data were concatenated and reduced by subject-level and group-level principal component analysis, using the Infomax algorithm. Subsequently, the GICA-3 back reconstruction step was used to separate single-subject components from the set of aggregate components. Finally, the spatial component maps were acquired and the value of connectivity strength within each IC was converted into a z-score.

After the ICA, the individual-level time courses of recognized components were deduced via the spatio-temporal double regression method. The relationship between the time courses of different pairwise networks was determined using static FNC analysis. Briefly, a band-pass filter (band-pass 0.01–0.15 Hz) was used to reduce the potential influence of low-and high-frequency noise on the time course. Then, the Pearson correlation coefficient between the ICs was performed to calculate the FNC strength. An FNC matrix with the dimensions of 12 times 12 (selected ICs) times 103 (participants) was realized.

### Prediction for non-steroidal anti-inflammatory drugs efficacy

Migraineurs were randomly divided into the training and testing cohorts, in the ratios of 80%/20%, 75%/25%, and 70%/30%, respectively. To classify the efficacy of NSAIDs, the SVM model based on abnormal FNC patterns between two migraine subgroups was trained by a fivefold cross-validation strategy in the training cohort. Subsequently, the model was tested on the internal testing cohort. The receiver operating characteristic (ROC) curve was plotted to determine the area under the curve (AUC). Calculations to determine sensitivity and specificity were also measured.

### Statistical analysis

The SPSS 25.0 software was used for statistical analyses and the level of significance was set at *p* < 0.05. The demographic characteristics of the three groups were compared using one-way analyses of variance (ANOVA) for the normally distributed continuous variables, while the Kruskal–Wallis test was employed for the non-normally distributed continuous variables. The clinical characteristics of the two migraine groups were compared using a two-sample *t*-test for the normally distributed continuous variables and the Mann–Whitney test for the non-normally distributed continuous variables. Comparisons between categorical variables were determined using the Chi-square test.

For FNC analysis, the correlation coefficients of each pairwise component among the three groups were compared. All *post hoc* tests were corrected by the Bonferroni correction method (*p* < 0.05/3). Furthermore, the partial correlation analysis between the FNC strength and migraine-related characteristics was calculated at a significant level of *p* < 0.05, controlling for age, sex, and education level.

## Results

### Demographics and clinical characteristics

After the fMRI data head motion check, three patients were excluded because of excessive head motion artifacts. Therefore, the final cohort consisted of patients with seventy MwoA and 33 healthy participants. There was no significant difference in the demographic and clinical data among the three groups (*p* > 0.05) (Table 1).

**TABLE 1:** The demographic and clinical characteristics of MwoA patients and HCs.

	M-eNSAIDs	M-ieNSAIDs	HCs	*F/t/χ 2*	*P*-value
Age (years)	33.91 ± 10.75	35.69 ± 11.47	36.30 ± 10.38	0.445	0.642
Sex (male/female)	3/32	4/31	2/31	0.669	0.907
Education (years)	13.54 ± 2.81	13.11 ± 3.08	12.39 ± 4.06	1.020	0.364
Disease duration (years)	7.94 ± 6.15	11.60 ± 10.08	/	–1.832	0.071
Frequency (days/month)	4.77 ± 3.15	5.23 ± 5.76	/	–0.412	0.682
Attack duration (hours)	17.31 ± 13.81	17.03 ± 15.05	/	0.083	0.934
VAS score	6.40 ± 1.54	5.97 ± 1.65	/	1.123	0.265
MIDAS score	38.97 ± 32.26	37.29 ± 31.78	/	0.220	0.826
HIT-6 score	57.57 ± 8.86	60.34 ± 9.41	/	–1.269	0.209
Drug types (Ibuprofen/Aspirin)	30/5	28/7	/	0.402	0.752

*Values for continuous variables are mean ± standard deviation. HCs, healthy controls; HIT, headache impact test; MIDAS, migraine disability assessment scale; M-eNSAIDs, MwoA with effective NSAIDs; M-ieNSAIDs, MwoA with ineffective NSAIDs; MwoA, migraine without aura; NSAIDs, non-steroid anti-inflammatory drugs; VAS, visual analogue scale.*

### Independent component analysis and component selection

**FIGURE 1:**
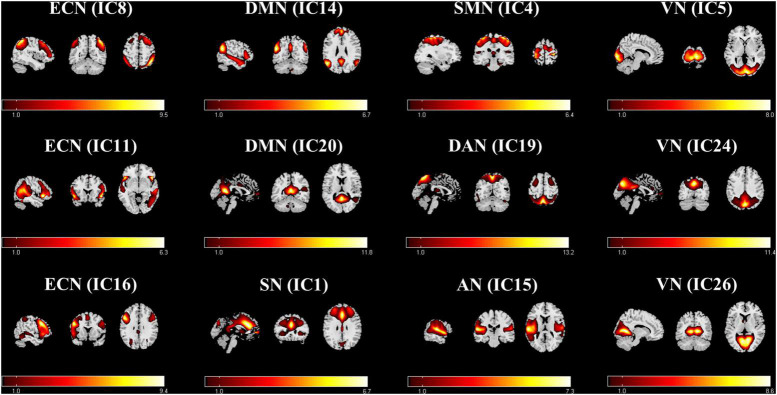
Functional relevant resting-state networks. The spatial maps of the 12 independent components were selected for further analysis. AN, auditory network; DAN, dorsal attention network; DMN, default mode network; ECN, executive control network; SMN, sensorimotor network; SN, salient network; VN, visual network.

In this study, 26 ICs were automatically extracted, and 12 components of them were selected as the resting-state networks for further analysis (Figure 1). Afterward, seven large-scale networks within the 12 components were labeled (Li et al., 2019, 2020). The first was the ECN (IC8, IC11, and IC16). This mainly focused on the dorsolateral prefrontal cortex, inferior parietal lobule, and superior parietal lobule. The second was the DMN (IC14, IC20), which includes the medial prefrontal cortex, posterior cingulate cortex (PCC), precuneus, inferior parietal gyrus, and angular gyrus. The third was the SN (IC1), which mainly consists of the anterior cingulate cortex (ACC), anterior insular cortex, and part of the prefrontal areas. The fourth was the sensorimotor network (SMN) (IC4), which included the supplementary motor area, the paracentral lobule, the precentral gyrus, and the postcentral gyrus. The fifth was the dorsal attention network (DAN) (IC19) which mainly includes the bilateral intraparietal sulcus, frontal eye field, and middle temporal lobe. The DAN was followed by the auditory network (AN) (IC15). The auditory network includes the temporal lobe and the surrounding temporal-parietal association cortex. Finally, was the visual network (VN) (IC5, IC24, IC26), which includes the primary visual cortex and extra-visual cortex.

### Group-level differences in functional network connectivity analysis

**FIGURE 2:**
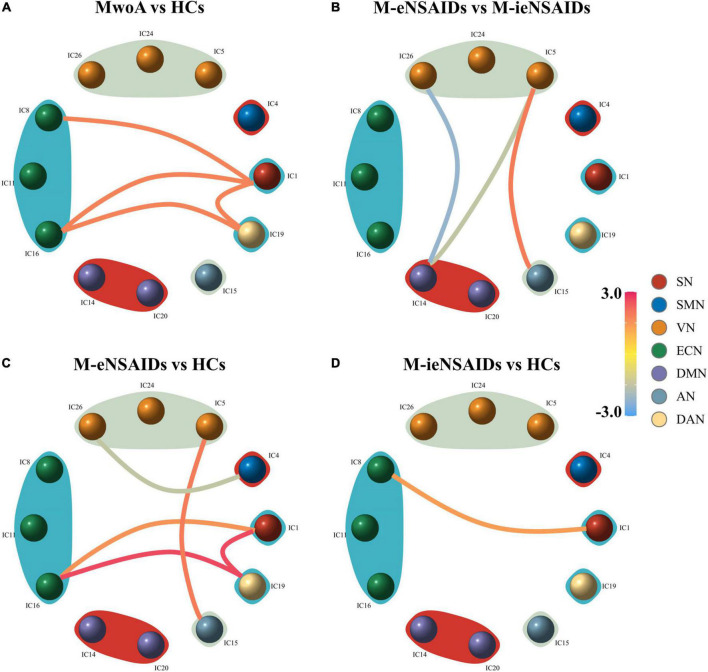
Group-level differences in static functional network connectivity patterns. **(A–D)** AN, auditory network; DAN, dorsal attention network; DMN, default mode network; ECN, executive control network; SMN, sensorimotor network; SN, salient network; VN, visual network; M-eNSAIDs, MwoA with effective NSAIDs; M-ieNSAIDs, MwoA with ineffective NSAIDs; MwoA, migraine without aura; NSAIDs, non-steroid anti-inflammatory drugs.

For the FNC analysis between all patients and HCs, patients with MwoA had increased connections, including SN (IC1)- ECN (IC8), SN (IC1)-ECN (IC16), SN (IC1)-DAN (IC19), and ECN (IC16)-DAN (IC19) (Figure 2A). For the FNC analysis among the three groups, nine FNC patterns were shown to be significantly altered (Figures 2B–D). Relative to patients with M-ieNSAIDs, patients with M-eNSAIDs exhibited significantly opposite interactions in three networks, including the decreased DMN (IC14)-VN (IC5, IC26) and increased VN (IC5)-AN (IC15) connections. Moreover, compared with HCs, patients with M-eNSAIDs showed significantly increased FNC patterns for SN (IC1)-ECN (IC16), SN (IC1)-DAN (IC19), VN (IC5)-AN (IC15), and ECN (IC16)-DAN (IC19) connections. On the contrary, there was revealed a decreased connection between SMN (IC4)-VN (IC26). In addition, SN (IC1)- ECN (IC8) connection was also found to be significantly increased in patients with M-ieNSAIDs, compared with HCs.

### Correlation analysis

**FIGURE 3:**
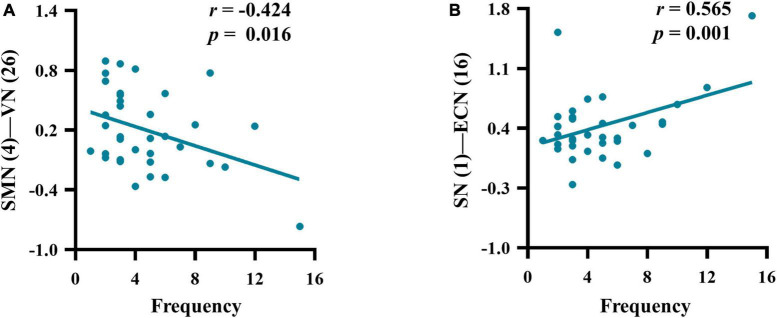
Correlations between the mean z-scores of functional network connectivity and headache frequency **(A–B)** in migraineurs with effective NSAIDs (for other abbreviations see Figure 2 legend).

There were significant correlations between the SMN-VN connection and frequency (*r* = −0.424, *p* = 0.016), as well as between the ECN-SN connection and frequency (*r* = 0.565, *p* = 0.001) in patients with M-eNSAIDs (Figure 3). However, there was no correlation between migraine-related characteristics and abnormal FNC patterns, neither in all patients with MwoA nor in patients who did not respond to NSAIDs.

### Support vector machine model performance

**FIGURE 4:**
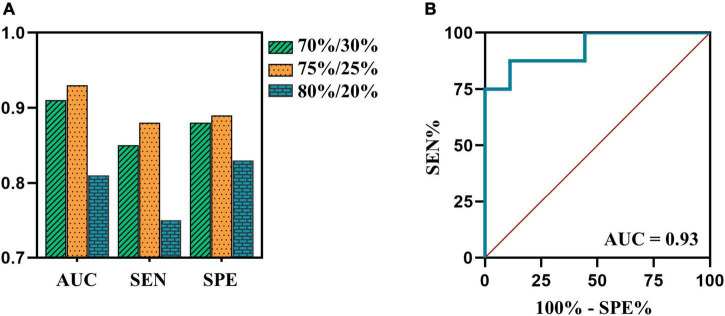
**(A)** The AUCs, sensitivity and specificity of SVM model in testing cohort of 70%/30%, 75%/25%, and 80%/20% datasets are 0.91, 0.85, and 0.88 (green bar); 0.93, 0.88, and 0.89 (orange bar); 0.81, 0.75, and 0.83 (blue bar), respectively. **(B)** The ROC curve of SVM model in testing cohort of 75%/25% dataset. AUC, area under the curve; ROC, receiver operating characteristic; SEN, sensitivity; SPE, specificity; SVM, support vector machine.

The SVM model with an allocation ratio of 75%/25% demonstrated a better predictive capacity, with an AUC of 0.93, the sensitivity of 0.88, and a specificity of 0.89 (Figure 4).

## Discussion

To our knowledge, this study is the first time through the ICA approach to explore the inter-network connectivity and the relationship with the efficacy of NSAIDs in patients with MwoA. The DMN and several somatosensory-associated networks, including the VN, AN, and SMN, were detected to be abnormal in patients with M-eNSAIDs. Meanwhile, the two migraine subgroups also showed increased alterations of the inter-network functional coupling in the higher-level executive areas, such as SN, ECN, and DAN, when compared with HCs. Interestingly, only SN-ECN functional abnormality was found between both two subgroups and HCs, which may indicate that the executive control function of the SN and ECN is mainly manifested in the potential neuroimaging features for migraine. These results reveal evidence of aberrant connectivity patterns across core neurocognitive networks in migraineurs and provide a proposition complementary to the point that migraine involves dysfunctional cortical-subcortical circuitry. Moreover, the observed correlations between FNC abnormalities and clinical parameters may not only improve the understanding of the pathophysiologic features of migraine but also further highlight neuroimaging characteristics in migraine treatment.

Visual and auditory discomforts are the most common complaints of migraine patients during both ictal and interictal periods. Thus, the visual and auditory cortex may play an important role in the neurophysiological mechanisms and treatment strategies. Moreover, the visual and auditory cortices have been demonstrated to be abnormal neural activity in the acupuncture treatment of migraine patients (Yang et al., 2012; Liu et al., 2020). An acupuncture study showed that the visual-related regions could predict the therapeutic efficacy of acupuncture in migraineurs without aura (Liu et al., 2020). In the present study, the functional connectivity strength of VN-AN in patients with M-eNSAIDs was significantly higher than that of patients with M-ieNSAIDs and HCs. It may imply that this connection pattern could play an important role in the development and treatment of migraine. Also, the pattern may be a neuroimaging marker for predicting the treatment efficacy for migraine. Moreover, a decreased FNC pattern between SMN and VN showed a significant negative correlation with headache frequency in the present study. Similarly, a prior study revealed that there was a bidirectional connection between SMN and VN in patients with MwoA (Wei et al., 2020). Furthermore, it was reported that the activity of SMN is positively correlated with headache frequency, which is contrary to our results. However, another study showed that multiple increased VN-related functional connections were negatively correlated with headache frequency in patients with MwoA (Wei et al., 2021). Although consistency has been maintained in the migraine population according to the ICHD-3, the controversial results of these studies may be caused by the patients of different subtypes and complex pathological mechanisms of migraine. Long-term and repeated migraine attacks could lead to somatosensory and visual cortex changes. When this happens, it may result in dysfunctional relief of pain and modulation of mental disorders, as well as the efficacy of NSAIDs. These findings suggest that altered intrinsic FNC architecture could provide a novel perspective to understand the neural circuits of NSAIDs in migraine, providing a more appropriate therapeutic strategy.

Results of the FNC abnormality of patients with M-eNSAIDs showed evidence of several prominent networks in the high cognitive and sensory networks. These included FNC pairs associated with DMN regions extending to sensory functional nodes (visual cortex and auditory cortex). The DMN is active in the resting condition and has a salient function in the functional network architecture of the resting-state brain. The main functions of the DMN are to maintain the steady state of the endogenous environment and exclude the interference of exogenous stimuli. When stimulated by endogenous or exogenous stimuli, the trigeminovascular pathway enters into an active status to suppress the DMN function and activates the executive regions to inhibit the pain signals. However, disrupted brain-network circuits contribute to inefficient control performance and headache attacks (Menon, 2019). A growing body of evidence (Zhang et al., 2016; Lo Buono et al., 2017) indicates similar results that, compared with HCs, migraine patients showed significantly increased spontaneous neuronal activity in the DMN. Also, the functional coupling between the DMN and other pain inhibitory cortex is associated with pain inhibition efficiency. Previous task-state fMRI studies (Hougaard et al., 2014; Wang et al., 2017) have reported greater visual-induced DMN activation in patients with migraine, indicating correlations and synchronizations between the DMN and VN. Hence, the disrupted DMN-VN connection may be involved in the information transfer and multimodal integration dysfunction affecting the clinical efficacy of NSAIDs for migraineurs.

A PET study (Di et al., 2013) also showed similar cerebral glucose hypermetabolism in the DMN in chronic migraine patients with analgesic overuse relative to patients without overuse. The ability of DMN to enhance functional hyperresponsiveness is possibly associated with pathogenesis and drug overuse. Similar to the aforementioned studies, the results of the current study reflected elevated functional connectivity in the static state involving the DMN in migraine patients. Additionally, Kong et al. (2010) studied the effect of pain intensity on DMN activation and deactivation in a healthy cohort, demonstrating an intriguing result that moderate-intensity nociception decreased DMN activity to a lesser extent than did severity-intensity nociception. The report suggested that deactivation of DMN may be a compensatory physiological response to the pain perception and higher intensity of painful stress exceeding the regulatory threshold of pain stimuli, leading to dysfunctional deactivation. On the basis of the current study, compared with the M-ieNSAIDs patients, only patients with M-eNSAIDs demonstrated lower connectivity strength between DMN and VN. These results suggest that NSAIDs may exert analgesic effects by depressing the activity of the DMN, but excessive DMN activity may eventually result in invalid efficacy. Nevertheless, an ASL study (Hodkinson et al., 2015) attended to explore the activation in response to NSAIDs-induced analgesia. The study concluded that there was significantly enhanced cerebral blood flow in the PCC and nearby limbic system, and significant negative analgesic interaction in the SN and prefrontal cortex in post-surgery patients. The completely opposite findings may be due to variations in methodology and heterogeneity of populations between post-surgery patients and headache-free migraineurs. On the other hand, similar to the current study, the ASL study verified that the functional imbalance of task-free and task-positive networks may be the substrate neuromechanism in the perception and modulation of pain.

The present findings further showed that enhanced FNC connections among the large-scale networks including ECN, DAN, and SN are important for efficient therapy. ECN and DAN are dubbed the “task-positive networks,” well-characterized by the performance of cognitive control and attention-demanding tasks. The previous study has called attention to the presence of anticorrelations between the DMN and the task-positive networks in pain perception (Ter Minassian et al., 2013). The study reported that during pain perception, the DMN was activated, as was the DAN, showing the anticorrelation between DMN and DAN disappears in chronic pain patients. This findings of this study are in agreement with our results that revealed lower DMN-related and higher task-related connections in migraineurs with an effective response to NSAID treatment. Moreover, the present study indicated that there was a significant increase in the connections between ECN-SN and DAN-SN. The SN, consisting of the insula and dorsal ACC, responds to the perception and chronification of pain (Lu et al., 2016), modulation of emotion (Gasquoine, 2014), and making of decisions (Chand and Dhamala, 2016). Furthermore, the SN, related to gating the nociceptive hypersensitivity pathway (Tan et al., 2017), performs its function as a switch to deactivate the DMN and activate the ECN when a salient stimulus is ongoing (Menon, 2019). These three components (DMN, ECN, and SN) constituted a “triple-network model,” mapping the salient aberrance and cognitive deficits across neurological disorders (Menon, 2011), including the migraine. Additionally, a nociceptive-task study displayed opposite functional patterns between the DMN and ECN observed in healthy subjects, confirming this functional coupling model is an effective physiological modulation for inhibiting nociception (Kong et al., 2010).

The results of this study showed similar anti-correlations among the three core networks, which may provide a new perspective on investigating the potential mechanisms underlying migraine treatment. Besides, the functional imbalance of the triple-network model in this study induced significant and potential correlations with headache frequency. Recently, gradually increasing evidence has suggested that clinical characteristics are strongly linked with inter-network functional deficits (Russo et al., 2012; Lo Buono et al., 2017; Coppola et al., 2018). Reciprocally, these migraine-related features may be potentially vital for influencing the progression of the disease and the quality of therapy (Zou et al., 2019). This result of association with headache frequency implies understanding of the neural mechanisms facilitating the transition from episodic to chronic migraine.

These results demonstrated that abnormal FNC patterns were associated with neurophysiological mechanisms of migraine and NSAID treatment, and represented a novelty approach to predicting NSAIDs efficacy, since to our knowledge network-level neuroimaging characteristics had never been considered predictive variables. We believe that our findings provide a foundation for a new line of research oriented to individually predicting NSAIDs efficacy in patients with MwoA, as well as its significant interaction with neuroimaging FNC characteristics. These neuroimaging variables could be taken into account for the selection and implementation of improving NSAIDs efficacy and enhancing the quality of life in patients with MwoA.

Although the study was to examine targeted relationships between FNC patterns and the efficacy of NSAIDs, some limitations need to be addressed. First, the large-scale brain networks involved in this study are limited. Other brain networks may also play a vital role in the neurophysiology of drug treatment in migraineurs, thus exploring the extending FNC abnormalities among the multiple networks may give insight into the neural mechanism. Second, cause-and-effect relationships between altered FNC patterns and the efficacy of NSAIDs should be cautiously interpreted using a longitudinal study in the future. Third, despite not taking any medication for 1 month before the scanning, the effect of other analgesic drugs on neurological function cannot be ruled out completely. Fourth, the models in this work underwent only internal tests, future work should focus on additional validation using external data to confirm the robustness. Moreover, no significant results persisted after Bonferroni correction for multiple comparisons in the FNC analyses due to the relatively strict method. Nonetheless, our research is still meaningful as it forms a basis upon which future novel studies can be developed. Finally, this study only explored the non-directional FNC interactions between static networks using the ICA approach. Further studies are needed to reflect the characteristics of temporal and directional function in the coupling between brain networks.

## Conclusion

The major strength and novelty of this study are that inter-static FNC patterns participate in the pain processing and medicine treatment in patients with migraine. The present data support the notion that the development of the antinociceptive effects of NSAIDs is mediated via the triple-network model, involving the descending pain inhibitory pathway, which provides meaningful insights for further understanding the neural mechanism of migraine treatment.

## Data availability statement

The original contributions presented in this study are included in the article/supplementary material, further inquiries can be directed to the corresponding author/s.

## Ethics statement

The studies involving human participants were reviewed and approved by the Ethics Committee of Nanjing Jiangning Hospital, affiliated with Nanjing Medical University. The patients/participants provided their written informed consent to participate in this study.

## Author contributions

H-LW, W-JY, and HZ designed the study. G-PZ and Y-SY acquired the data. H-LW, Y-CC, and Y-SY performed the data analysis. W-JY, Y-CC, and XY interpreted the results. H-LW and W-JY prepared the manuscript. JL and HZ revised the manuscript and corrected the English. All authors contributed to manuscript revision and approved the submitted version.

## Footnotes

1
http://restfmri.net/forum/

2
http://icatb.sourceforge.net/

## References

[B1] Abrams E. M. Akombi B. Alam S. Alcalde-Rabanal J. E. Allebeck P. Amini-Rarani M. (2020). Global burden of 369 diseases and injuries in 204 countries and territories, 1990–2019: A systematic analysis for the Global Burden of Disease Study 2019. *Lancet* 396 1204–1222. 10.1016/S0140-6736(20)30925-9 33069326 PMC7567026

[B2] Ashina M. (2020). Migraine. *New Engl. J. Med*. 383 1866–1876. 10.1056/NEJMra1915327 33211930

[B3] Brennan K. C. Pietrobon D. (2018). A systems neuroscience approach to migraine. *Neuron* 97 1004–1021. 10.1016/j.neuron.2018.01.029 29518355 PMC6402597

[B4] Chand G. B. Dhamala M. (2016). The salience network dynamics in perceptual decision-making. *Neuroimage* 134 85–93. 10.1016/j.neuroimage.2016.04.018 27079535

[B5] Chen C. Yan M. Yu Y. Ke J. Xu C. Guo X. (2019). Alterations in regional homogeneity assessed by fMRI in patients with migraine without aura. *J. Med. Syst*. 43:298. 10.1007/s10916-019-1425-z 31352647

[B6] Coppola G. Di Renzo A. Tinelli E. Di Lorenzo C. Scapeccia M. Parisi V. (2018). Resting state connectivity between default mode network and insula encodes acute migraine headache. *Cephalalgia* 38 846–854. 10.1177/0333102417715230 28605972

[B7] Di W. Shi X. Zhu Y. Tao Y. Qi W. Luo N. (2013). Overuse of paracetamol caffeine aspirin powders affects cerebral glucose metabolism in chronic migraine patients. *Eur. J. Neurol*. 20 655–662. 10.1111/ene.12018 23114018

[B8] Feigin V. L. Nichols E. Alam T. Bannick M. S. Beghi E. Blake N. (2019). Global, regional, and national burden of neurological disorders, 1990–2016: A systematic analysis for the Global Burden of Disease Study 2016. *Lancet Neurol*. 18 459–480. 10.1016/S1474-4422(18)30499-X 30879893 PMC6459001

[B9] Gasquoine P. G. (2014). Contributions of the insula to cognition and emotion. *Neuropsychol. Rev*. 24 77–87. 10.1007/s11065-014-9246-9 24442602

[B10] Goadsby P. J. Holland P. R. Martins-Oliveira M. Hoffmann J. Schankin C. Akerman S. (2017). Pathophysiology of migraine: A disorder of sensory processing. *Physiol. Rev*. 97 553–622. 10.1152/physrev.00034.2015 28179394 PMC5539409

[B11] Headache Classification Committee of the International Headache Society [IHS] (2013). The International Classification of Headache Disorders, 3rd edition (beta version). *Cephalalgia* 33 629–808. 10.1177/0333102413485658 23771276

[B12] Hodkinson D. J. Khawaja N. O’Daly O. Thacker M. A. Zelaya F. O. Wooldridge C. L. (2015). Cerebral analgesic response to nonsteroidal anti-inflammatory drug ibuprofen. *Pain* 156 1301–1310. 10.1097/j.pain.0000000000000176 25851460

[B13] Hougaard A. Amin F. M. Hoffmann M. B. Rostrup E. Larsson H. B. W. Asghar M. S. (2014). Interhemispheric differences of fMRI responses to visual stimuli in patients with side-fixed migraine aura. *Hum. Brain Mapp*. 35 2714–2723. 10.1002/hbm.22361 24038870 PMC6869529

[B14] Kong J. Loggia M. L. Zyloney C. Tu P. LaViolette P. Gollub R. L. (2010). Exploring the brain in pain: Activations, deactivations and their relation. *Pain* 148 257–267. 10.1016/j.pain.2009.11.008 20005043 PMC2815185

[B15] Kwon J. Lee H. Cho S. Chung C. Lee M. J. Park H. (2020). Machine learning-based automated classification of headache disorders using patient-reported questionnaires. *Sci. Rep.* 10:14062. 10.1038/s41598-020-70992-1 32820214 PMC7441379

[B16] Li C. Deng Y. He Y. Zhai H. Jia F. (2019). The development of brain functional connectivity networks revealed by resting-state functional magnetic resonance imaging. *Neural Regen. Res*. 14:1419. 10.4103/1673-5374.253526 30964068 PMC6524509

[B17] Li F. Lu L. Shang S. Hu L. Chen H. Wang P. (2020). Disrupted functional network connectivity predicts cognitive impairment after acute mild traumatic brain injury. *CNS Neurosci. Ther*. 26 1083–1091. 10.1111/cns.13430 32588522 PMC7539836

[B18] Liu M. Gao Y. Sun G. Dong M. Yin T. Tian Z. (2020). The spontaneous activity pattern of the middle occipital gyrus predicts the clinical efficacy of acupuncture treatment for migraine without aura. *Front. Neurol*. 11:588207. 10.3389/fneur.2020.588207 33240209 PMC7680874

[B19] Lo Buono V. Bonanno L. Corallo F. Pisani L. R. Lo Presti R. Grugno R. (2017). Functional connectivity and cognitive impairment in migraine with and without aura. *J. Headache Pain* 18:72. 10.1186/s10194-017-0782-6 28730563 PMC5519515

[B20] Lu C. Yang T. Zhao H. Zhang M. Meng F. Fu H. (2016). Insular cortex is critical for the perception, modulation, and chronification of pain. *Neurosci. Bull*. 32 191–201. 10.1007/s12264-016-0016-y 26898298 PMC5563738

[B21] Mennini F. S. Gitto L. Martelletti P. (2008). Improving care through health economics analyses: Cost of illness and headache. *J. Headache Pain* 9 199–206. 10.1007/s10194-008-0051-9 18604472 PMC3451939

[B22] Menon B. (2019). Towards a new model of understanding -The triple network, psychopathology and the structure of the mind. *Med. Hypotheses* 133:109385. 10.1016/j.mehy.2019.109385 31494485

[B23] Menon V. (2011). Large-scale brain networks and psychopathology: A unifying triple network model. *Trends Cogn. Sci*. 15 483–506. 10.1016/j.tics.2011.08.003 21908230

[B24] Mu J. Chen T. Quan S. Wang C. Zhao L. Liu J. (2020). Neuroimaging features of whole-brain functional connectivity predict attack frequency of migraine. *Hum. Brain Mapp*. 41 984–993. 10.1002/hbm.24854 31680376 PMC7267923

[B25] Pérez Benito F. J. Conejero J. A. Sáez C. García Gómez J. M. Navarro Pardo E. Florencio L. L. (2019). Subgrouping factors influencing migraine intensity in women: A semi-automatic methodology based on machine learning and information geometry. *Pain Pract*. 20 297–309. 10.1111/papr.12854 31677218

[B26] Russo A. Tessitore A. Giordano A. Corbo D. Marcuccio L. De Stefano M. (2012). Executive resting-state network connectivity in migraine without aura. *Cephalalgia* 32 1041–1048. 10.1177/0333102412457089 22908362

[B27] Schwedt T. J. Chiang C. Chong C. D. Dodick D. W. (2015). Functional MRI of migraine. *Lancet Neurol*. 14 81–91. 10.1016/S1474-4422(14)70193-0 25496899 PMC11318354

[B28] Tan L. L. Pelzer P. Heinl C. Tang W. Gangadharan V. Flor H. (2017). A pathway from midcingulate cortex to posterior insula gates nociceptive hypersensitivity. *Nat. Neurosci*. 20 1591–1601. 10.1038/nn.4645 28920932

[B29] Ter Minassian A. Ricalens E. Humbert S. Duc F. Aubé C. Beydon L. (2013). Dissociating anticipation from perception: Acute pain activates default mode network. *Hum. Brain Mapp*. 34 2228–2243. 10.1002/hbm.22062 22438291 PMC6870109

[B30] Tu Y. Zeng F. Lan L. Li Z. Maleki N. Liu B. (2020). An fMRI-based neural marker for migraine without aura. *Neurology* 94 e741–e751. 10.1212/WNL.0000000000008962 31964691 PMC7176301

[B31] Wang M. Su J. Zhang J. Zhao Y. Yao Q. Zhang Q. (2017). Visual cortex and cerebellum hyperactivation during negative emotion picture stimuli in migraine patients. *Sci. Rep.* 7:41919. 10.1038/srep41919 28181500 PMC5299401

[B32] Wei H. Chen J. Chen Y. Yu Y. Guo X. Zhou G. (2020). Impaired effective functional connectivity of the sensorimotor network in interictal episodic migraineurs without aura. *J. Headache Pain* 21:111. 10.1186/s10194-020-01176-5 32928098 PMC7489040

[B33] Wei H. Li J. Guo X. Zhou G. Wang J. Chen Y. (2021). Functional connectivity of the visual cortex differentiates anxiety comorbidity from episodic migraineurs without aura. *J. Headache Pain* 22:40. 10.1186/s10194-021-01259-x 34020591 PMC8138918

[B34] Yadav M. P. Ballal S. Meckel M. Roesch F. Bal C. (2020). [177Lu]Lu-DOTA-ZOL bone pain palliation in patients with skeletal metastases from various cancers: Efficacy and safety results. *EJNMMI Res*. 10 1–13. 10.1186/s13550-020-00709-y 33113035 PMC7593375

[B35] Yang J. Zeng F. Feng Y. Fang L. Qin W. Liu X. (2012). A PET-CT study on the specificity of acupoints through acupuncture treatment in migraine patients. *BMC Complement. Altern. Med*. 12:123. 10.1186/1472-6882-12-123 22894176 PMC3480944

[B36] Yu D. Yuan K. Luo L. Zhai J. Bi Y. Xue T. (2017). Abnormal functional integration across core brain networks in migraine without aura. *Mol. Pain* 13:19341042. 10.1177/1744806917737461 28969471 PMC5644367

[B37] Zhang J. Su J. Wang M. Zhao Y. Yao Q. Zhang Q. (2016). Increased default mode network connectivity and increased regional homogeneity in migraineurs without aura. *J. Headache Pain* 17:98. 10.1186/s10194-016-0692-z 27771875 PMC5075323

[B38] Zou Y. Tang W. Li X. Xu M. Li J. (2019). Acupuncture reversible effects on altered default mode network of chronic migraine accompanied with clinical symptom relief. *Neural Plast*. 2019 1–10. 10.1155/2019/5047463 31011330 PMC6442315

